# Muscle Atrophy‐Related Adverse Events of Antidiabetic Drug Classes: A Pharmacovigilance Analysis Using VigiBase Data

**DOI:** 10.1002/jcsm.70251

**Published:** 2026-04-14

**Authors:** Shiho Ueta, Takahiro Niimura, Mitsuhiro Goda, Tomoaki Ishida, Naohiro Iwata, Tatsuaki Takeda, Kei Kawada, Koji Miyata, Fuka Aizawa, Kenta Yagi, Hirofumi Hamano, Masayuki Chuma, Yuki Izawa‐Ishizawa, Yoshito Zamami, Keisuke Ishizawa

**Affiliations:** ^1^ Department of Clinical Pharmacology and Therapeutics University of Tokushima Graduate School of Biomedical Sciences Tokushima Japan; ^2^ Clinical Research Center for Developmental Therapeutics Tokushima University Hospital Tokushima Japan; ^3^ Department of Clinical Pharmacology and Therapeutics, Graduate School of Biomedical and Heath Sciences Hiroshima University Hiroshima Japan; ^4^ Department of Clinical Pharmacy, Graduate School of Pharmaceutical Sciences Nagoya City University Nagoya Japan; ^5^ Department of Pharmacy Okayama University Hospital Okayama Japan; ^6^ Department of Pharmacy Tokushima University Hospital Tokushima Japan; ^7^ Department of Pharmacy Shimane University Hospital Izumo Shimane Japan; ^8^ Department of Hospital Pharmacy and Pharmacology Asahikawa Medical University Asahikawa Japan; ^9^ Department of Health and Nutrition, Faculty of Human Life Science Shikoku University Tokushima Japan

**Keywords:** antidiabetic drug, muscle atrophy, sarcopenia, VigiBase

## Abstract

**Background:**

Diabetes mellitus—a chronic metabolic disorder associated with an increased risk of muscle atrophy—can significantly impact patients' quality of life and overall health outcomes. While antidiabetic medications are crucial for managing blood glucose levels, some have been linked to muscle‐related adverse events, potentially exacerbating the already elevated risk of muscle deterioration in diabetic patients. However, a comprehensive analysis of muscle atrophy‐related adverse events across different classes of antidiabetic drugs has been lacking. Therefore, this study investigates the profile of muscle atrophy‐related adverse events across major antidiabetic drug classes using the World Health Organization's (WHO's) Individual Case Safety Reports database.

**Methods:**

A pharmacovigilance analysis was conducted using data from VigiBase, the WHO's global reporting database, from 1968 to September 2025. The study examined adverse event signals related to muscle atrophy, sarcopenia, muscular weakness and motor function decline for nine classes of antidiabetic medications. Reporting odds ratios (RORs) were calculated to assess signal detection, and co‐occurrence patterns of adverse events were analysed.

**Results:**

Among 41 551 306 adverse event reports, 2 095 847 were related to antidiabetic medications. Safety signals for muscle atrophy were detected with sulfonylureas (ROR: 1.2, 95% CI: 1.01–1.43, *p* = 0.042), GLP‐1 analogues (ROR: 1.2, 95% CI: 1.02–1.41, *p* = 0.031) and SGLT2 inhibitors (ROR: 1.5, 95% CI: 1.19–1.78, *p* < 0.001). SGLT2 inhibitors also showed a signal for sarcopenia (ROR: 6.2, 95% CI: 3.71–10.3, *p* < 0.001). Biguanides demonstrated signals for muscular weakness (ROR: 1.6, 95% CI: 1.54–1.71, *p* < 0.001) and motor function decline (ROR: 1.7, 95% CI: 1.41–2.13, *p* < 0.001). Thiazolidinediones, glinides, DPP‐4 inhibitors and alpha‐glucosidase inhibitors showed no safety signals for the examined adverse events. Additionally, co‐occurrence analysis revealed frequent associations between muscle atrophy and nausea/vomiting, falls and decreased appetite across different drug classes.

**Conclusions:**

These findings indicate notable differences in the profiles of muscle atrophy–related adverse events among major classes of antidiabetic drugs, suggesting that drug selection may influence the risk of muscle function decline in patients. Clinicians should consider these safety profiles when prescribing antidiabetic therapies; however, causal relationships cannot be inferred solely from pharmacovigilance data. Further studies are warranted to establish causality between antidiabetic drug use and muscle‐related adverse events and to elucidate the underlying mechanisms.

## 1 Introduction

Diabetes mellitus is recognized as a significant risk factor for muscle atrophy [1, 2]. Chronic hyperglycemia and insulin resistance associated with diabetes lead to enhanced protein degradation and suppressed protein synthesis, resulting in decreased muscle mass and function. Consequently, muscle atrophy in diabetic patients is acknowledged as a critical complication that can lead to reduced physical function and quality of life. Skeletal muscle is a primary target organ of insulin, and a reduction in skeletal muscle mass has been reported to impair glucose metabolism [3]. This bidirectional relationship between diabetes and skeletal muscle mass reduction creates a vicious cycle where each condition worsens the other.

In recent years, adverse events such as muscle atrophy and muscle weakness have been reported in association with multiple antidiabetic agents, particularly SGLT2 inhibitors [4, 5, 6]. Since diabetic patients often require long‐term antidiabetic medication, the effects of these agents on skeletal muscle warrant careful evaluation. However, conflicting effects of antidiabetic drugs on muscle have been reported. A clinically important consideration is whether the observed reductions in muscle mass result from direct pharmacological effects on skeletal muscle tissue or are secondary to drug‐induced weight loss. This distinction is crucial for understanding the clinical significance of these observations. For example, while some studies report that GLP‐1 receptor agonists exert beneficial effects on muscle, long‐term clinical trials have documented “disproportionate” reductions in lean body mass in patients receiving these agents [6, 7]. Given such clinical complexity, the current knowledge gaps regarding the effects of individual antidiabetic agents on skeletal muscle underscore the need for further detailed investigation.

However, comprehensive comparative studies examining the incidence and characteristics of muscle atrophy‐related adverse events across different classes of diabetes medications have not yet been conducted. Consequently, the disparities in adverse event profiles concerning muscle atrophy, sarcopenia and muscle weakness among various antidiabetic drug classes remain unclear.

This study investigates the profile of muscle atrophy‐related adverse events associated with major antidiabetic medications using the Individual Case Safety Reports (ICSRs) database of the World Health Organization (WHO). Furthermore, it explains the concomitant symptoms reported alongside these adverse events. This analysis elucidates the characteristics of muscle atrophy‐related adverse events for each antidiabetic drug class to derive insights for developing safer diabetes treatment.

## 2 Methods

### 2.1 Pharmacovigilance Analysis

VigiBase is the largest global reporting database for medicinal products maintained by the Uppsala Monitoring Centre in Sweden, with over 40 million adverse event reports collected since 1968 (Uppsala Monitoring Centre, VigiBase). Data from 1968 to September 2025 were used in this study. As VigiBase contains duplicate reports, we excluded duplicate reports using statistical algorithms developed by the Uppsala Monitoring Center. The downloaded data were processed using the SQLite database (version 3.33.0; SQLite Consortium, Charlotte, NC, USA). This study was approved by the Ethics Committee of Tokushima University Hospital (approval number: 4492).

The association of antidiabetic drugs with reports of various muscle atrophy‐related adverse events was investigated. The following drugs were included in the analysis (Table S1): thiazolidinediones [ATC code: A10BG], sulfonylureas [ATC code: A10BB], SGLT2 inhibitors [ATC code: A10BK], GLP‐1 analogues [ATC code: A10BJ], DPP‐4 inhibitors [ATC code: A10BH], biguanides [ATC code: A10BA], insulins and analogues [ATC code: A10A], alpha‐glucosidase inhibitors [ATC code: A10BF] and glinides [ATC code: A10BX02 repaglinide, A10BX03 nateglinide and A10BX08 mitiglinide]. Tirzepatide was not included in the analysis of the present study. The definitions of adverse events in this study adhered to those in the Medical Dictionary for Regulatory Activities (MedDRA), developed by the International Conference on Harmonization of Technical Requirements for the Registration of Pharmaceuticals for Human Use (ICH, MedDRA). Each adverse event is listed in Table S2.

Further, we calculated and evaluated the frequency of co‐reported adverse event combinations to investigate the associations between various muscle atrophy‐related adverse events and complications. We performed this step to elucidate potential correlations among simultaneously occurring adverse events.

### 2.2 Statistical Analysis

Categorical variables were expressed as frequencies and percentages. Statistical significance was set at *p* < 0.05. A safety signal was defined as information suggesting a new or known adverse event that may be associated with a medicinal product and warrants further evaluation [8, 9]. Safety signal detection was assessed using reporting odds ratios (RORs). A signal was considered present when the lower limit of the 95% confidence interval (CI) for the ROR exceeded 1 [10]. An ROR is defined as the odds ratio of the drug of interest regarding a specific adverse event report. Adverse event reports were divided into four groups: (a) those reporting the adverse drug reaction (ADR) of the drug of interest; (b) those not reporting the ADR of the drug of interest; (c) those reporting the ADR of all other drugs; and (d) those not reporting the ADR of all other drugs. The ROR was calculated as follows:

$$\mathrm{ROR}=\left(a/b\right)/\left(c/d\right)$$

The combinations of adverse events reported simultaneously were identified by calculating the reporting proportions of other adverse events for each adverse event. This analysis was visualized using a chord diagram. Additionally, we employed the Ω shrinkage measure—a method for detecting drug–drug interactions—to evaluate the interaction between biguanides and GLP‐1 analogues, between biguanides and SGLT2 inhibitors, as well as between GLP‐1 analogues and SGLT2 inhibitors. An interaction was deemed present if the lower bound of the 95% CI for Ω exceeded zero [11, 12]. These analyses were performed using R statistical software (version 4.0.3; R Foundation, Vienna, Austria) [13]. Data processing was conducted using the R packages dplyr (ver. 1.1.4) and tidyr (ver. 1.3.1). Baseline characteristics were summarized with tableone (ver. 0.13.2), while graphical representations were created using ggplot2 (ver. 3.5.2) and circlize (ver. 0.4.16).

## 3 Results

The analysed data comprised 41 551 306 adverse event reports, of which 2 095 847 were related to antidiabetic medications (Table S3).

**FIGURE 1:**
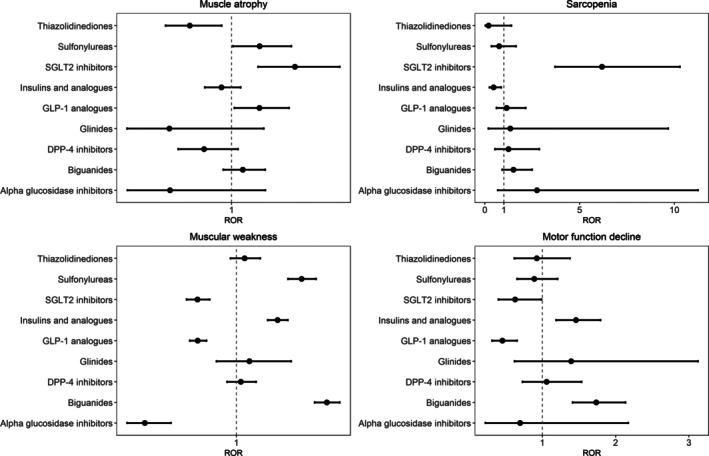
ROR total. RORs and 95% confidence intervals for each drug are illustrated. The central line indicates the reference value of ROR = 1. DPP‐4 inhibitor, dipeptidyl peptidase‐4 inhibitor; GLP‐1 analogue, glucagon‐like peptide‐1 analogue; ROR, reporting odds ratio; SGLT2 inhibitor, sodium‐glucose cotransporter 2 inhibitor.

Analysis of adverse event signals revealed distinct patterns across different medication classes (Figure 1). For muscle atrophy, safety signals were detected with sulfonylureas (ROR: 1.2, 95% CI: 1.01–1.43, *p* = 0.042), SGLT2 inhibitors (ROR: 1.5, 95% CI: 1.19–1.78, *p* < 0.001) and GLP‐1 analogues (ROR: 1.2, 95% CI: 1.02–1.41, *p* = 0.031). Regarding sarcopenia, signals were observed with SGLT2 inhibitors (ROR: 6.2, 95% CI: 3.71–10.3, *p* < 0.001). Reports of muscular weakness showed association with sulfonylureas (ROR: 1.5, 95% CI: 1.36–1.55, *p* < 0.001), insulin and analogues (ROR: 1.3, 95% CI: 1.22–1.35, *p* < 0.001) and biguanides (ROR: 1.6, 95% CI: 1.54–1.71, *p* < 0.001), while motor function decline demonstrated signals with insulin and analogues (ROR: 1.5, 95% CI: 1.19–1.79, *p* < 0.001) and biguanides (ROR: 1.7, 95% CI: 1.41–2.13, *p* < 0.001). Conversely, thiazolidinediones, glinides, DPP‐4 inhibitors and alpha‐glucosidase inhibitors showed no safety signals for any of the examined adverse events.

**FIGURE 2:**
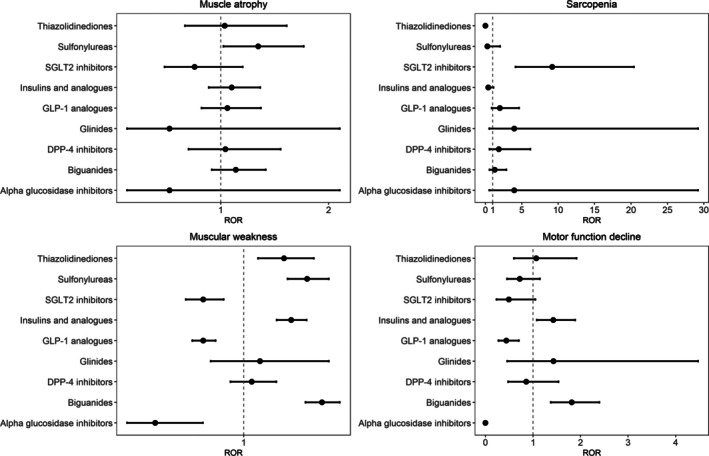
ROR female. RORs and 95% confidence intervals for each drug are illustrated. The central line indicates the reference value of ROR = 1. DPP‐4 inhibitor, dipeptidyl peptidase‐4 inhibitor; GLP‐1 analogue, glucagon‐like peptide‐1 analogue; ROR, reporting odds ratio; SGLT2 inhibitor, sodium‐glucose cotransporter 2 inhibitor.

**FIGURE 3:**
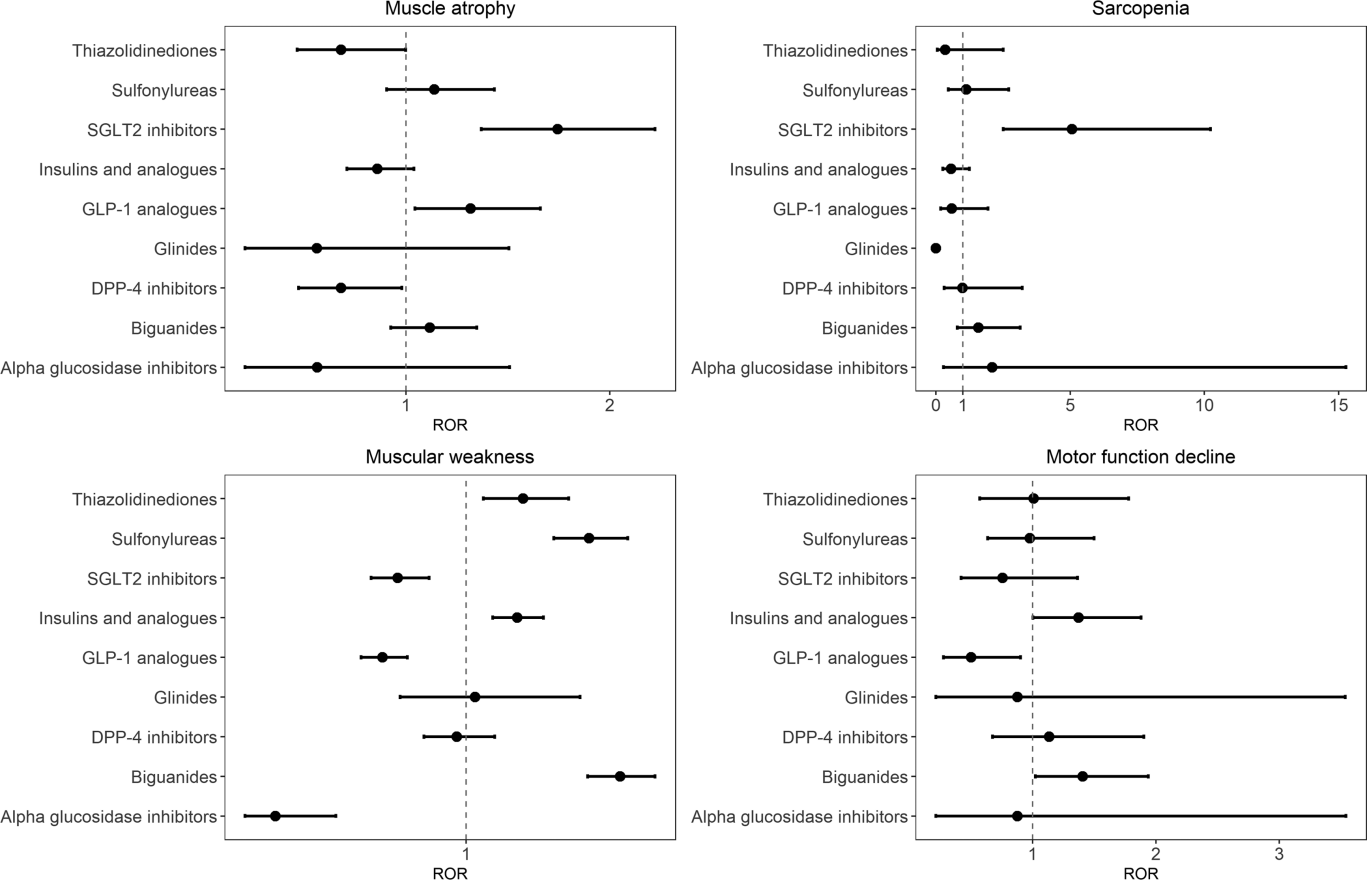
ROR male. RORs and 95% confidence intervals for each drug are illustrated. The central line indicates the reference value of ROR = 1. DPP‐4 inhibitor, dipeptidyl peptidase‐4 inhibitor; GLP‐1 analogue, glucagon‐like peptide‐1 analogue; ROR, reporting odds ratio; SGLT2 inhibitor, sodium‐glucose cotransporter 2 inhibitor.

The analysis of adverse events associated with various antidiabetic medications revealed sex‐specific differences in reporting frequency. The results for females and males are visualized in Figures 2 and 3, respectively. In females, biguanide users showed significantly high frequency for muscular weakness (ROR: 1.5, 95% CI: 1.39–1.61, *p* < 0.001) and motor function decline (ROR: 1.8, 95% CI: 1.38–2.4, *p* < 0.001). SGLT2 inhibitor users demonstrated a high ROR for sarcopenia (ROR: 9.2, 95% CI: 4.12–20.41, *p* < 0.001). In males, biguanide users showed significantly high frequency for muscular weakness (ROR: 1.6, 95% CI: 1.48–1.75, *p* < 0.001) and motor function decline (ROR: 1.4, 95% CI: 1.02–1.94, *p* = 0.039). Male SGLT2 inhibitor users exhibited significantly high RORs for muscle atrophy (ROR: 1.7, 95% CI: 1.37–2.22, *p* < 0.001) and sarcopenia (ROR: 5.1, 95% CI: 2.51–10.23, *p* < 0.001).

**FIGURE 4:**
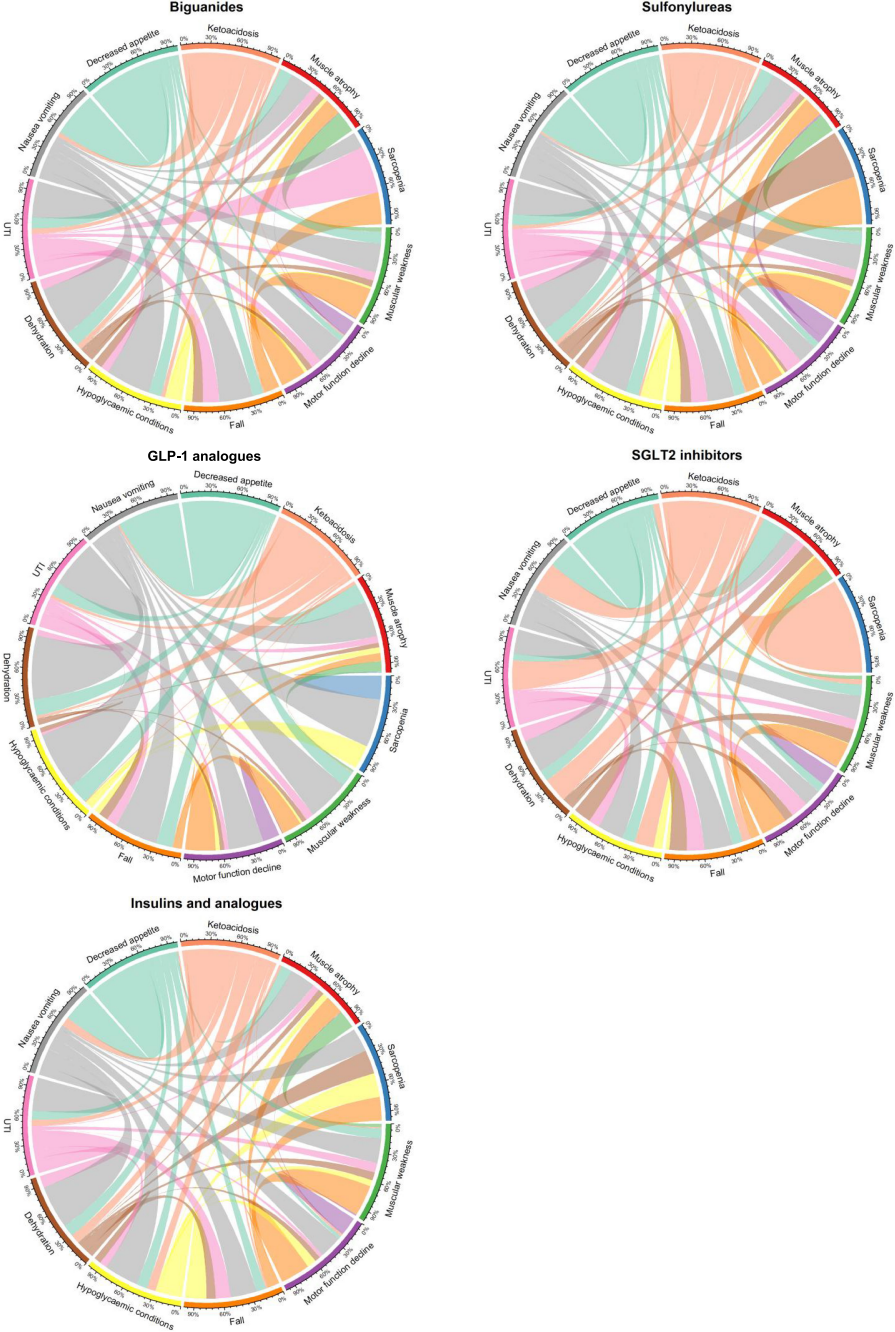
Combinations of adverse events reported simultaneously. Chord diagram illustrating the interrelationships among comorbidities. The width of each arc represents the frequency of co‐occurrence between the respective adverse events in users of each drug. GLP‐1 analogue, glucagon‐like peptide‐1 analogue; SGLT2 inhibitor, sodium‐glucose cotransporter 2 inhibitor; UTI, urinary tract infection.

Moreover, analysis of adverse event co‐occurrence patterns among biguanide users revealed several notable relationships (Figure 4). Among 291 cases of muscle atrophy, frequent co‐occurrences were observed with nausea/vomiting (50 cases) and falls (35 cases). Motor function decline (157 cases) and muscular weakness (2277 cases) showed similar patterns, with falls being the most common co‐occurring condition (22 and 375 cases, respectively). A total of 61 838 cases of nausea/vomiting were reported, with 4458 cases accompanied by decreased appetite and 1634 cases accompanied by dehydration.

Furthermore, analysis of adverse event co‐occurrence patterns among sulfonylurea users revealed noticeable relationships. Among 152 cases of muscle atrophy, co‐occurrences were most frequent with nausea/vomiting (29 cases) and falls (22 cases). Motor function decline (47 cases) and muscular weakness (1069 cases) showed relatively low co‐occurrence rates, primarily with falls (7 and 200 cases, respectively). A total of 22 347 cases of nausea/vomiting were reported, with 2052 cases accompanied by decreased appetite and 741 cases accompanied by dehydration. Notably, hypoglycemic conditions (15 793 cases) showed substantial co‐occurrence with falls (239 cases) and nausea/vomiting (465 cases).

Among SGLT2 inhibitor users, distinct patterns of adverse event co‐occurrences were observed. Among 108 cases of muscle atrophy, co‐occurrences were most frequent with decreased appetite (11 cases) and nausea/vomiting (six cases). Motor function decline (20 cases) and muscular weakness (348 cases) showed limited co‐occurrences with other conditions. A total of 8795 cases of nausea/vomiting were reported, with 555 cases accompanied by decreased appetite and 411 cases accompanied by dehydration.

The drug–drug interactions between biguanides and either GLP‐1 analogues or SGLT2 inhibitors were analysed with respect to the adverse event of muscle atrophy using the Ω statistic (Table 1). The results are presented in Table 1. The Ω was −0.15 (95% CI: −0.61–0.32) for GLP‐1 analogues and 0.25 (95% CI: −0.23–0.73) for SGLT2 inhibitors. Further, the Ω was 0.33 (95% CI: −0.40–1.06) for the combination of GLP‐1 analogues and SGLT2 inhibitors; in both cases, the lower bounds of the 95% CIs did not exceed 0, indicating the absence of a statistically significant drug interaction for muscle atrophy adverse events between biguanides and these two classes of antidiabetic medications.

**TABLE 1:** Evaluation of drug–drug interactions.

Drug 1	Drug 2	Adverse event	Ω	95% CI min of Ω
Biguanides	GLP‐1 analogues	Muscle atrophy	−0.15	−0.61
Biguanides	SGLT2 inhibitors	Muscle atrophy	0.25	−0.23
GLP‐1 analogues	SGLT2 inhibitors	Muscle atrophy	0.33	−0.40

Abbreviations: 95% CI, 95% confidence interval; GLP‐1 analogue, glucagon‐like peptide 1 analogue; SGLT2 inhibitor, sodium‐glucose cotransporter 2 inhibitor.

## 4 Discussion

This study investigated the profile of muscle atrophy‐related adverse events associated with major antidiabetic medications and elucidated the concomitant symptoms reported alongside these events. Our analysis of the WHO's ICSRs database revealed distinct patterns of muscle atrophy‐related adverse events across different antidiabetic drug classes. Notably, sulfonylureas, SGLT2 inhibitors and GLP‐1 analogues showed safety signals for muscle atrophy, with SGLT2 inhibitors demonstrating an association with reports of sarcopenia. Conversely, thiazolidinediones, glinides, DPP‐4 inhibitors and alpha‐glucosidase inhibitors did not show safety signals for the examined adverse events. These findings provide valuable insights into the differential effects of antidiabetic medications on muscle health, which is crucial for optimizing diabetes treatment strategies.

The findings of this study demonstrate safety signals for muscle‐related adverse events across several antidiabetic drug classes, aligning with previous studies while also presenting novel insights. The observed association between SGLT2 inhibitors and reports of muscle atrophy as well as sarcopenia is consistent with recent literature suggesting that SGLT2 inhibitors may lead to reductions in lean body mass and skeletal muscle mass [4, 5]. This effect has been attributed to the activation of gluconeogenesis and promotion of lipolysis, potentially facilitating the breakdown of skeletal muscle proteins [4]. Conversely, although safety signals related to muscle atrophy were detected for sulfonylureas and GLP‐1 analogues in our study, several studies have reported that these drug classes either attenuate muscle atrophy or exert no appreciable effect on muscle mass [6]. The lower limit of the 95% confidence interval observed in our study was close to unity, indicating that these findings should be interpreted cautiously and warrant further investigation before any definitive conclusions can be drawn.

The present study also revealed an intriguing difference in safety signal profiles among antidiabetic agents. Structural changes in muscle mass, such as atrophy, were detected as safety signals associated with SGLT2 inhibitors, whereas functional alterations in muscle performance—such as muscle weakness and motor function decline—were observed with insulin and its analogues as well as biguanides. Functional impairments of skeletal muscles (e.g., reduced strength or motor performance) typically require a longer time to manifest than structural changes in muscle volume. Moreover, muscle strength is influenced not only by skeletal muscle mass but also by neural function. Considering these temporal discrepancies in symptom onset and the complex underlying pathophysiology, our findings underscore the need for careful clinical evaluation of these effects and for further studies to elucidate their mechanistic basis.

The analysis of adverse event co‐occurrence patterns among users of different antidiabetic medications revealed distinct safety profiles and potential mechanistic insights. Reports of muscle atrophy associated with various medications were frequently reported in conjunction with adverse events such as nausea/vomiting and decreased appetite. Indeed, gastrointestinal adverse events have been documented in association with sulfonylureas, GLP‐1 analogues and biguanides [14, 15, 16]. Reportedly, metformin, a biguanide class medication, increases the population of *Escherichia coli* in the intestinal tract. This phenomenon has been associated with an elevated release of lipopolysaccharides, leading to gastrointestinal side effects such as abdominal distension and diarrhoea. These observations highlight the potential mechanistic link between metformin‐induced alterations in gut microbiota and the manifestation of adverse gastrointestinal symptoms [16]. These gastrointestinal symptoms may impede patients' nutritional intake, potentially leading to deficiencies in nutrients essential for maintaining muscle mass. While the use of antidiabetic drugs may improve glycemic control, the gastrointestinal side effects of these drugs can result in inadequate nutritional intake, potentially increasing the risk of skeletal muscle atrophy. This clinical observation underscores the importance of nutritional interventions in diabetic patients that focus on improving blood glucose levels and maintaining skeletal muscle mass. Additionally, there was a tendency for adverse events of muscle atrophy and falls to be reported simultaneously with sulfonylureas. Notably, a reduction in muscle mass can increase the risk of falls [17]; therefore, it is necessary to develop strategies to mitigate fall risk associated with muscle atrophy in patients receiving antidiabetic medications.

Diabetes treatment typically commences with monotherapy and progresses to combination therapy if the initial approach proves insufficient. Metformin, a biguanide, has long been the first‐line pharmacological intervention for uncomplicated type 2 diabetes mellitus due to its cost‐effectiveness and favourable safety profile [18]. In recent years, the therapeutic landscape has expanded to include SGLT2 inhibitors, GLP‐1 analogues and DPP‐4 inhibitors, which are now widely prescribed based on individual patient characteristics and comorbidities [19]. This study assessed the impact of combination therapy using biguanides in conjunction with either SGLT2 inhibitors or GLP‐1 analogues. The analysis of drug interactions for muscle atrophy adverse events using Ω revealed no significant interactions between biguanides and either SGLT2 inhibitors or GLP‐1 analogues. However, the sample size for these combination therapies was limited, and the RORs exhibited wide CIs. Consequently, we cannot definitively conclude whether the concomitant use of these agents with biguanides exerts a beneficial effect on skeletal muscle. Nevertheless, these findings underscore the necessity for further investigation into the impact of combination antidiabetic therapies on muscle atrophy. Future studies should elucidate the potential synergistic or antagonistic effects of these drug combinations on muscle health in patients with diabetes mellitus.

This study suggests that the use of antidiabetic drugs may have differential effects on skeletal muscle based on gender. Specifically, SGLT2 inhibitor use was associated with reports of muscle atrophy in males, while no such association was observed in females. While hormonal influences play a significant role in gender differences in drug effect, previous research has reported that SGLT2 inhibitor administration does not affect levels of male hormones such as testosterone [20]. Currently, there is limited clear evidence regarding the direct impact of SGLT2 inhibitors on estrogen. From an energy metabolism perspective, studies have reported differences between males and females in amino acid utilization during exercise, indicating that males may more actively metabolize amino acids under conditions of restricted glucose utilization [21]. These findings collectively highlight the complex interplay between gender, pharmacological interventions and metabolic responses, emphasizing the need for gender‐specific considerations in diabetes management and further research to elucidate the underlying mechanisms of these observed differences. Males generally have greater muscle mass and higher levels of growth hormone and testosterone secretion compared with females. Given these physiological differences, rapid weight loss and energy depletion induced by SGLT2 inhibitors may create conditions that promote muscle breakdown, potentially leading to a more pronounced reduction in muscle mass, particularly in males [22, 23].

This study has several research limitations. The first limitation concerns the potential inconsistency in the diagnostic criteria for muscle atrophy. The methods for evaluating muscle atrophy are diverse, including bioelectrical impedance analysis, computed tomography (CT) and magnetic resonance imaging (MRI) techniques [24, 25]. Moreover, the evaluation standards proposed vary by region. VigiBase, the database used in this study, covers a global population and includes temporal variations. Consequently, the evaluation criteria may not be uniform across the adverse event reports. The second limitation is the inability to account for the effects of nonpharmacological interventions such as exercise therapy and dietary interventions [26, 27]. These nondrug treatments are known to influence skeletal muscle strength and function. Therefore, it is possible that such factors have impacted the results of this study. Third, although the findings of this study may inform future clinical decision‐making, the current dataset did not include detailed treatment information, such as the prescribed dosages or therapeutic courses of antidiabetic agents, nor does it contain data on the rationale underlying each treatment decision. The lack of such prescription‐level clinical background information represents an important limitation of this study. To adequately address these potential confounding factors and establish a causal relationship between the use of antidiabetic drugs and muscle‐related outcomes, further research employing detailed clinical datasets is warranted. The fourth limitation concerns the lack of detailed data on treatment responsiveness. Specifically, information on changes in body weight and blood glucose levels associated with the use of each antidiabetic agent was unavailable. Such information is crucial for understanding the pathophysiological progression of muscle atrophy, as changes in body weight are directly linked to both the progression of muscle wasting and overall prognosis. Among antidiabetic medications, SGLT2 inhibitors and GLP‐1 receptor agonists are known to induce weight loss. Because these agents may influence muscle mass and function, the absence of data on fluctuations in body weight and blood glucose levels represents an important limitation in the interpretation of the study findings.

In conclusion, this comprehensive pharmacovigilance analysis of the WHO's ICSRs database has revealed significant differences in muscle atrophy‐related adverse event profiles among various antidiabetic medication classes. Sulfonylureas, SGLT2 inhibitors and GLP‐1 analogues demonstrated safety signals for muscle atrophy. The complication analysis further elucidated the complex interplay between muscle‐related adverse events and other symptoms, particularly highlighting the frequent association with nausea/vomiting, falls and decreased appetite across different drug classes. These findings provide valuable insights into the differential impacts of antidiabetic medications on muscle health. However, while some of the RORs estimated in this study may suggest potential safety signals, their clinical significance is likely limited. Given the exploratory nature of this analysis, additional investigations are needed to confirm the clinical relevance and applicability of these observations.

## Funding

This work was supported by JST SPRING, Grant Number JPMJSP2113.

## Ethics Statement

This study was approved by the Ethics Committee of Tokushima University Hospital (approval number: 4492).

## Conflicts of Interest

The authors declare no conflicts of interest.

## Supporting information


**Table S1:** Definition of drug classification.
**Table S2:** Definition of adverse event classification.
**Table S3:** Characteristics of each drug user group.

## Data Availability

The data supporting the findings of this study are available from VigiBase with permission from the Uppsala Monitoring Center and used under licence; therefore, they are not publicly available.
