# Supplementary material for: Muscle Atrophy‐Related Adverse Events of Antidiabetic Drug Classes: A Pharmacovigilance Analysis Using VigiBase Data
Source: J Cachexia Sarcopenia Muscle. 2026 Apr 14;17(2):e70251. doi: 10.1002/jcsm.70251 (PMC13078952; doi:10.1002/jcsm.70251)
Supplement: Supplementary file 1 — Table S1: Definition of drug classification. Table S2: Definition of adverse event classification. Table S3: Characteristics of each drug user group. [file JCSM-17-e70251-s001.docx]

Supplementary Table 1. Definition of Drug Classification

| Drug group | ATC code | General name |
| --- | --- | --- |
| Thiazolidinediones | A10BG | Lobeglitazone |
|  | A10BG | Pioglitazone |
|  | A10BG | Rivoglitazone |
|  | A10BG | Rosiglitazone |
| Sulfonylureas | A10BB | Acetohexamide |
|  | A10BB | Carbutamide |
|  | A10BB | Chlorpropamide |
|  | A10BB | Glibenclamide |
|  | A10BB | Glibornuride |
|  | A10BB | Gliclazide |
|  | A10BB | Glimepiride |
|  | A10BB | Glipalamide |
|  | A10BB | Glipizide |
|  | A10BB | Gliquidone |
|  | A10BB | Glisentide |
|  | A10BB | Glisolamide |
|  | A10BB | Glisoxepide |
|  | A10BB | Glyclopyramide |
|  | A10BB | Glycyclamide |
|  | A10BB | Metahexamide |
|  | A10BB | Tolazamide |
|  | A10BB | Tolbutamide |
| SGLT2 inhibitors | A10BK | Bexagliflozin |
|  | A10BK | Canagliflozin |
|  | A10BK | Dapagliflozin |
|  | A10BK | Empagliflozin |
|  | A10BK | Enavogliflozin |
|  | A10BK | Ertugliflozin |
|  | A10BK | Ipragliflozin |
|  | A10BK | Luseogliflozin |
|  | A10BK | Remogliflozin |
|  | A10BK | Sotagliflozin |
|  | A10BK | Tofogliflozin |
| GLP-1 analog | A10BJ | Albiglutide |
|  | A10BJ | Dulaglutide |
|  | A10BJ | Efpeglenatide |
|  | A10BJ | Exenatide |
|  | A10BJ | Liraglutide |
|  | A10BJ | Lixisenatide |
|  | A10BJ | Loxenatide |
|  | A10BJ | Semaglutide |
|  | A10BJ | Taspoglutide |
| DPP4 inhibitors | A10BH | Alogliptin |
|  | A10BH | Anagliptin |
|  | A10BH | Denagliptin |
|  | A10BH | Dutogliptin |
|  | A10BH | Evogliptin |
|  | A10BH | Gemigliptin |
|  | A10BH | Linagliptin |
|  | A10BH | Omarigliptin |
|  | A10BH | Saxagliptin |
|  | A10BH | Sitagliptin |
|  | A10BH | Teneligliptin |
|  | A10BH | Trelagliptin |
|  | A10BH | Vildagliptin |
| Alpha glucosidase inhibitors | A10BF | Acarbose |
|  | A10BF | Miglitol |
|  | A10BF | Voglibose |
| Glinides | A10BX | Mitiglinide |
|  | A10BX | Nateglinide |
|  | A10BD | Repaglinide |
|  | A10BX | Repaglinide |

Supplementary Table S2. Definition of Adverse Event Classification

| **Adverse event group** | **MedDRA Id** |
| --- | --- |
| Muscle atrophy | 10028289 |
|  | 10002027 |
| Sarcopenia | 10063024 |
| Muscular weakness | 10082799 |
|  | 10028373 |
|  | 10062556 |
| Motor function decline | 10061296 |
|  | 10082636 |
|  | 10071033 |
| Fall | 10016173 |
| Hypoglycaemic conditions | 10020993 |
|  | 10020994 |
|  | 10020997 |
|  | 10021000 |
|  | 10021002 |
|  | 10022472 |
|  | 10040576 |
|  | 10048803 |
|  | 10054998 |
|  | 10059035 |
|  | 10060378 |
|  | 10061211 |
|  | 10065981 |
|  | 10077010 |
|  | 10077216 |
|  | 10080024 |
|  | 10082152 |
|  | 10082172 |
|  | 10083495 |
| Dehydration | 10012174 |
| Urinary tract infections (UTI) | 10011781 |
|  | 10023424 |
|  | 10034531 |
|  | 10037584 |
|  | 10037596 |
|  | 10037597 |
|  | 10037601 |
|  | 10037653 |
|  | 10038351 |
|  | 10046424 |
|  | 10046470 |
|  | 10046480 |
|  | 10046571 |
|  | 10046573 |
|  | 10049100 |
|  | 10051250 |
|  | 10051959 |
|  | 10052299 |
|  | 10056351 |
|  | 10058596 |
|  | 10061182 |
|  | 10061395 |
|  | 10066757 |
|  | 10068822 |
|  | 10072058 |
|  | 10074457 |
|  | 10078229 |
|  | 10078604 |
|  | 10082153 |
|  | 10084121 |
|  | 10087963 |
| Nausea　vomiting | 10020614 |
|  | 10027183 |
|  | 10027975 |
|  | 10028813 |
|  | 10038776 |
|  | 10047700 |
|  | 10047708 |
|  | 10047709 |
|  | 10058938 |
|  | 10059256 |
|  | 10062937 |
|  | 10063338 |
|  | 10063559 |
|  | 10064670 |
|  | 10066220 |
|  | 10066962 |
|  | 10066963 |
|  | 10067171 |
|  | 10075315 |
|  | 10079120 |
|  | 10083633 |
| Decreased appetite | 10061428 |
| Ketoacidosis | 10012671 |
|  | 10023379 |

Supplementary Table 3. Characteristics of each drug user group

|  | Biguanides | Sulfonylureas | Thiazolidinediones | DPP4 inhibitor | GLP1 analogues | SGLT2 inhibitor | Insulins and analogues | Alpha GI | Glinide |
| --- | --- | --- | --- | --- | --- | --- | --- | --- | --- |
| n | 624718 | 294687 | 163616 | 161373 | 350974 | 174472 | 703005 | 24279 | 24489 |
| AgeGroup |  |  |  |  |  |  |  |  |  |
| 0 - 27 days | 280 (0.0) | 78 (0.0) | 25 (0.0) | 33 (0.0) | 132 (0.0) | 67 (0.0) | 1163 (0.2) | 13 (0.1) | 8 (0.0) |
| 28 days to 23 months | 199 (0.0) | 149 (0.1) | 18 (0.0) | 35 (0.0) | 92 (0.0) | 62 (0.0) | 608 (0.1) | 7 (0.0) | 6 (0.0) |
| 2 - 11 years | 385 (0.1) | 186 (0.1) | 53 (0.0) | 34 (0.0) | 112 (0.0) | 56 (0.0) | 7517 (1.1) | 14 (0.1) | 8 (0.0) |
| 12 - 17 years | 1556 (0.2) | 156 (0.1) | 63 (0.0) | 41 (0.0) | 860 (0.2) | 68 (0.0) | 7841 (1.1) | 29 (0.1) | 20 (0.1) |
| 18 - 44 years | 50778 (8.1) | 13847 (4.7) | 7985 (4.9) | 6171 (3.8) | 30434 (8.7) | 10028 (5.7) | 63955 (9.1) | 1274 (5.2) | 759 (3.1) |
| 45 - 64 years | 218548 (35.0) | 93906 (31.9) | 54071 (33.0) | 41884 (26.0) | 101976 (29.1) | 49803 (28.5) | 188225 (26.8) | 7899 (32.5) | 6003 (24.5) |
| 65 - 74 years | 138289 (22.1) | 72118 (24.5) | 27982 (17.1) | 36766 (22.8) | 50913 (14.5) | 32346 (18.5) | 127443 (18.1) | 6677 (27.5) | 6397 (26.1) |
| >= 75 years | 85936 (13.8) | 57660 (19.6) | 14755 (9.0) | 36681 (22.7) | 19065 (5.4) | 23524 (13.5) | 92715 (13.2) | 6582 (27.1) | 7697 (31.4) |
| Unknown | 128747 (20.6) | 56587 (19.2) | 58664 (35.9) | 39728 (24.6) | 147390 (42.0) | 58518 (33.5) | 213538 (30.4) | 1784 (7.3) | 3591 (14.7) |
| Sex |  |  |  |  |  |  |  |  |  |
| Female | 337343 (54.0) | 145487 (49.4) | 64034 (39.1) | 75534 (46.8) | 210254 (59.9) | 74536 (42.7) | 365936 (52.1) | 11470 (47.2) | 11472 (46.8) |
| Male | 265960 (42.6) | 138980 (47.2) | 72312 (44.2) | 77846 (48.2) | 121488 (34.6) | 84390 (48.4) | 305630 (43.5) | 12406 (51.1) | 12426 (50.7) |
| Unknown | 21415 (3.4) | 10220 (3.5) | 27270 (16.7) | 7993 (5.0) | 19232 (5.5) | 15546 (8.9) | 31439 (4.5) | 403 (1.7) | 591 (2.4) |
| Region |  |  |  |  |  |  |  |  |  |
| African Region | 5673 (0.9) | 2595 (0.9) | 90 (0.1) | 537 (0.3) | 420 (0.1) | 1338 (0.8) | 5670 (0.8) | 10 (0.0) | 7 (0.0) |
| Region of the Americas | 333461 (53.4) | 153986 (52.3) | 142742 (87.2) | 65087 (40.3) | 255917 (72.9) | 88792 (50.9) | 439672 (62.5) | 2483 (10.2) | 7965 (32.5) |
| South-East Asia Region | 21327 (3.4) | 12547 (4.3) | 1932 (1.2) | 6886 (4.3) | 900 (0.3) | 3354 (1.9) | 23100 (3.3) | 1374 (5.7) | 147 (0.6) |
| European Region | 161255 (25.8) | 67477 (22.9) | 7828 (4.8) | 31864 (19.7) | 72098 (20.5) | 51689 (29.6) | 148962 (21.2) | 4096 (16.9) | 9997 (40.8) |
| Eastern Mediterranean Region | 22488 (3.6) | 8298 (2.8) | 527 (0.3) | 5538 (3.4) | 5175 (1.5) | 4934 (2.8) | 22391 (3.2) | 75 (0.3) | 153 (0.6) |
| Western Pacific Region | 80514 (12.9) | 49784 (16.9) | 10497 (6.4) | 51461 (31.9) | 16464 (4.7) | 24365 (14.0) | 63210 (9.0) | 16241 (66.9) | 6220 (25.4) |
| Adverse event |  |  |  |  |  |  |  |  |  |
| Muscle atrophy | 291 (0.0) | 152 (0.1) | 58 (0.0) | 58 (0.0) | 180 (0.1) | 108 (0.1) | 295 (0.0) | 6 (0.0) | 6 (0.0) |
| Sarcopenia | 25 (0.0) | 7 (0.0) | 1 (0.0) | 6 (0.0) | 12 (0.0) | 23 (0.0) | 12 (0.0) | 2 (0.0) | 1 (0.0) |
| Muscular weakness | 2277 (0.4) | 1069 (0.4) | 448 (0.3) | 442 (0.3) | 718 (0.2) | 348 (0.2) | 2194 (0.3) | 24 (0.1) | 71 (0.3) |
| Motor function decline | 157 (0.0) | 47 (0.0) | 26 (0.0) | 30 (0.0) | 31 (0.0) | 20 (0.0) | 157 (0.0) | 3 (0.0) | 6 (0.0) |
